# Three-dimensional anatomical characterization of the sustentaculum tali in asymptomatic adults: a computed tomography-based morphometric analysis

**DOI:** 10.3389/fsurg.2025.1644876

**Published:** 2025-11-20

**Authors:** Runnan Xue, Yayun Sun, Wenfeng Zhu

**Affiliations:** Department of Orthopedics, Affiliated Hospital 6 of Nantong University (Yancheng Third People's Hospital), Yancheng, China

**Keywords:** sustentaculum tali, calcaneus, talus joint, anatomy, three-dimensional image

## Abstract

**Purpose:**

This study aimed to establish normative anatomical references for the sustentaculum tali and talar articular surface using three-dimensional CT imaging, and to develop clinically applicable diagnostic criteria for sustentaculum tali dislocation.

**Methods:**

We analyzed 104 normal calcanei (47 males/57 females; mean age 38.96 ± 12.97 years; 51 left/53 right feet) from January to August 2023. Three-dimensional calcaneal reconstructions were created using CT imaging data. Eight anatomical parameters were measured: sustentaculum tali dimensions (length/width/height), angular parameters (α: superior sustentaculum angle; β: inferior talar margin angle; γ: inclination angle between anterior articular surface projection and calcaneal plantar axis), and posterior talar space distance (L). Statistical comparisons were performed between genders and sides.

**Result:**

Morphometric analysis revealed the following anatomical parameters of the sustentaculum tali: length 36.93 ± 4.30 mm, width 14.65 ± 1.96 mm, and height 17.91 ± 2.34 mm. Angular measurements demonstrated *α* = 107.74 ± 9.59°, *β* = 115.34 ± 9.41°, and *γ* = 52.67 ± 5.98°, with posterior talar gap L measuring 4.83 ± 0.56 mm. Gender-based comparisons showed statistically significant differences in three-dimensional sustentaculum tali morphology (length/width/height) and posterior space L (all *P* < 0.001), with female measurements being consistently smaller than males. No significant inter-gender differences were observed in angular parameters (α/β/γ, *P* > 0.0). Laterality analysis revealed no statistically meaningful variations between left and right feet across all measured parameters.

**Conclusion:**

This study establishes normative 3D-CT anatomical parameters of the sustentaculum tali in healthy individuals (*n* = 104), revealing significant gender-based differences in dimensions and posterior talar gap distance L (all *P* < 0.001). Critical gender-specific diagnostic thresholds for dislocation were defined: *L* > 7.25 mm (males) and >6.49 mm (females). Combined with screw trajectory angles (α/β/γ) and sustentaculum dimensions (length/width/height), these data provide an evidence-based framework for preoperative planning, urgent reduction indication, and precision screw placement in calcaneal fracture management.

## Introduction

The calcaneus is a principal weight-bearing bone within the human skeletal system. As the most susceptible tarsal bone to trauma, fractures of the calcaneus constitute approximately 2% of all osseous fractures encountered in clinical practice. Moreover, it is estimated that around 75% of these calcaneal fractures involve the subtalar joint (1). The prognosis for fractures involving the subtalar articular surface is notably suboptimal, with a higher incidence of disability compared to fractures at other anatomic locations. Additionally, the outcomes of treatment for these fractures are generally less favorable (2). Currently, the treatment modality widely accepted by the majority of scholars for intra-articular fractures is open reduction and internal fixation, which is regarded as the “gold standard” in the management of such fractures (3, 4). In the reduction and fixation of calcaneal fractures involving the subtalar joint via a lateral approach, it is frequently necessary to reference the medial superior fragment of the fracture, which maintains a normal anatomical relationship with the talus (5).

The Sustentaculum tali of the calcaneus constitutes a critical anatomical landmark, situated between the calcaneal body and the articular surface of the talus. It plays a pivotal role in bearing and transmitting forces directed from the talus (6). Owing to the robust protection afforded by the surrounding ligaments, joint capsule, and tendons, the Sustentaculum tali is less prone to displacement in the event of a calcaneal fracture (7, 8). Consequently, the Sustentaculum tali serves as a reliable reference and a point of fixation for the reduction of calcaneal fractures, and it constitutes an exemplary anatomical landmark for the restoration of the calcaneal articular surface. Given the Sustentaculum tali's stable architecture and its high cortical bone density, it is commonly utilized as a site for screw placement in the surgical management of calcaneal fractures (9, 10). Precision in the placement of screws through the lateral wall into the Sustentaculum tali is instrumental in effectively preserving the integrity of the subtalar joint. Furthermore, it facilitates the restoration of biomechanical stability and the strength of the calcaneus following a calcaneal fracture (11). Historically, it was believed that the intra-articular talar bone fragment experienced minimal displacement due to the preservation of the calcaneal and deltoid ligaments. However, an increasing body of research has substantiated that calcaneal fractures in conjunction with talar fractures, dislocations, or subluxations occur with a significant frequency (12–14) that pose challenges in treatment. The investigation into the normal anatomical characteristics of the sustentaculum tali and the calcaneal joint, along with the quantification of 3D imaging data via 3D three-dimensional, holds considerable significance. Despite the critical role of the sustentaculum tali in calcaneal fracture management, comprehensive quantitative criteria for diagnosing its dislocation remain undefined, particularly gender-specific thresholds. Furthermore, optimal screw placement—essential for avoiding complications—lacks standardized guidance based on 3D anatomical parameters. To address these gaps, this study aimed to: (1) Quantify normative anatomical parameters of the sustentaculum tali (length, width, height, angles α/β/γ, and posterior talar gap L) in healthy adults using 3D-CT; (2) Compare these parameters by gender and laterality; (3) Establish evidence-based, gender-specific diagnostic thresholds for sustentaculum tali dislocation to guide surgical reduction and screw placement. Utilizing 3D-CT scans from 104 normal calcanei (January–August 2023), we provide foundational data for improving clinical outcomes in calcaneal fracture treatment.

## Materials and methods

### Criteria for the inclusion and exclusion of research subjects

Criteria inclusion: ① Aged between 18 and 70 years, regardless of gender or foot laterality; ② Individuals with normal foot appearance and CT findings.

Criteria exclusion: ① Presence of ankle or talus fractures affecting joint function; ② Underlying conditions impacting ankle joint integrity, such as diabetic foot or thromboangiitis obliterans; ③ Congenital foot and ankle deformities; ④ Neuromuscular diseases.

### General information

This study is of a descriptive nature. Adhering to the predefined criteria, a total of 104 participants with normal 3D three-dimensional scans of the foot and ankle were enrolled from January 2023 to August 2023 at the Affiliated Hospital 6 of Nantong University. The cohort comprised 47 males (involving 47 feet) and 57 females (involving 57 feet), with a mean age of (38.96 ± 12.97) years. The distribution included 51 feet from the left side and 53 feet from the right side.

### Methods

The foot and ankle underwent scanning using the American GE64 128-slice spiral CT (Light-speed VCT) scanner. The scanning parameters included a tube voltage of 120 V, current of 250 mA, three-dimensional reconstruction interval of 0.625 mm, slice thickness of 5 mm, and data processing workstation software Powervision6.0. 3D images of the calcaneus were reconstructed and analyzed.

### Anatomical measurements of the sustentaculum tali

**Figure 1:**
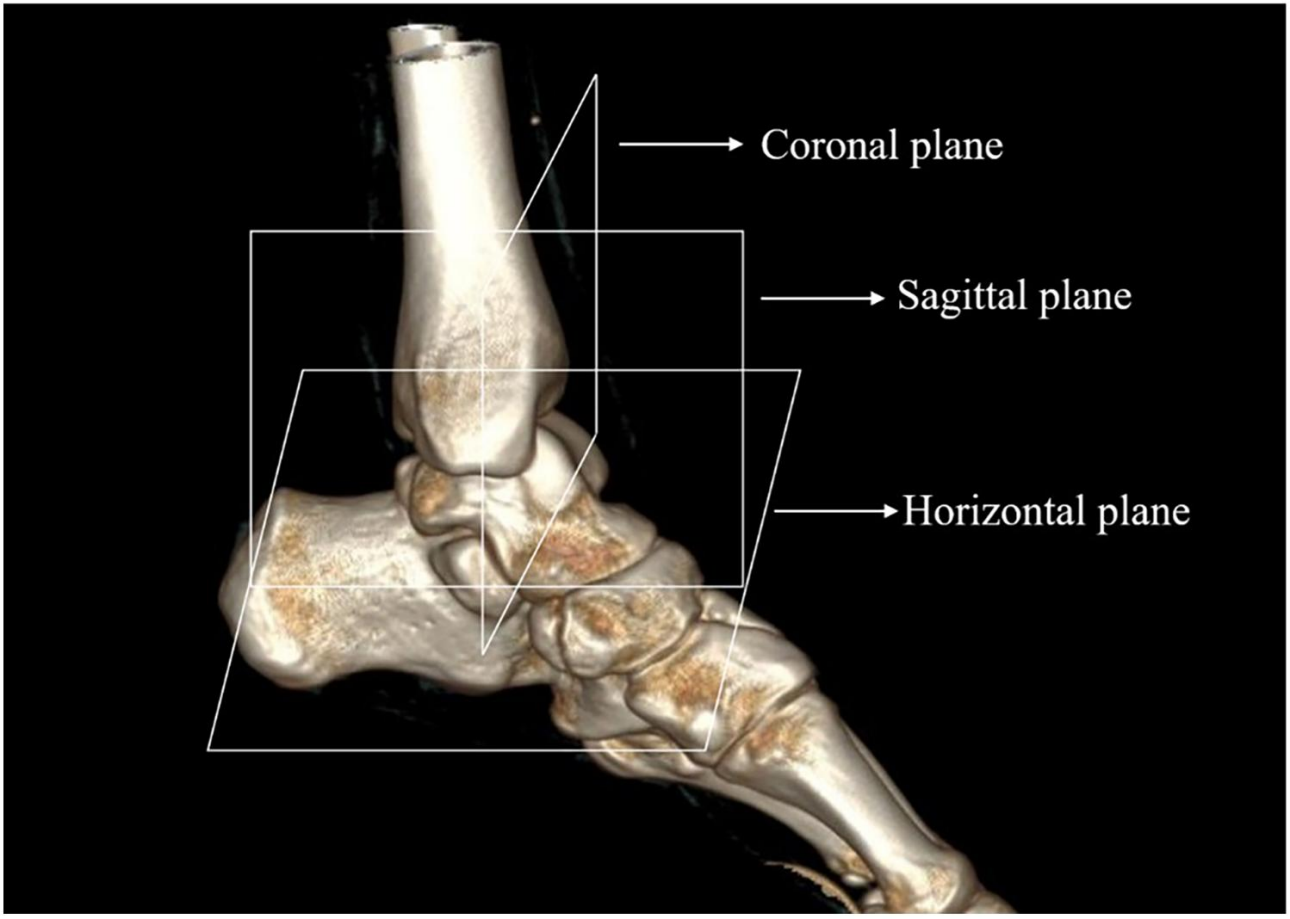
The reference plane of the ankle. Each reference plane is determined in the anteroposterior and lateral positions of the foot to maintain the ankle in a neutral functional position, without dorsiflexion or plantarflexion, valgus or varus deviation, or any deviation of the plantar plane. The sagittal reference plane is parallel to the medial wall of the foot and perpendicular to the horizontal reference plane, while the coronal reference plane is perpendicular to both of these planes.

Establishment of individual reference planes: With the ankle maintained in a functional position—neither in dorsiflexion nor plantarflexion, devoid of valgus or varus deformity, and ensuring the plantar plane is oriented without any deviation—the plane corresponding to the plantar surface is designated as the horizontal reference plane. The plane that is parallel to the medial aspect of the foot and perpendicular to the horizontal reference plane is identified as the sagittal reference plane. Concurrently, the plane that is orthogonal to both the established horizontal and sagittal reference planes is termed the coronal reference plane (Figure 1).

**Figure 2:**
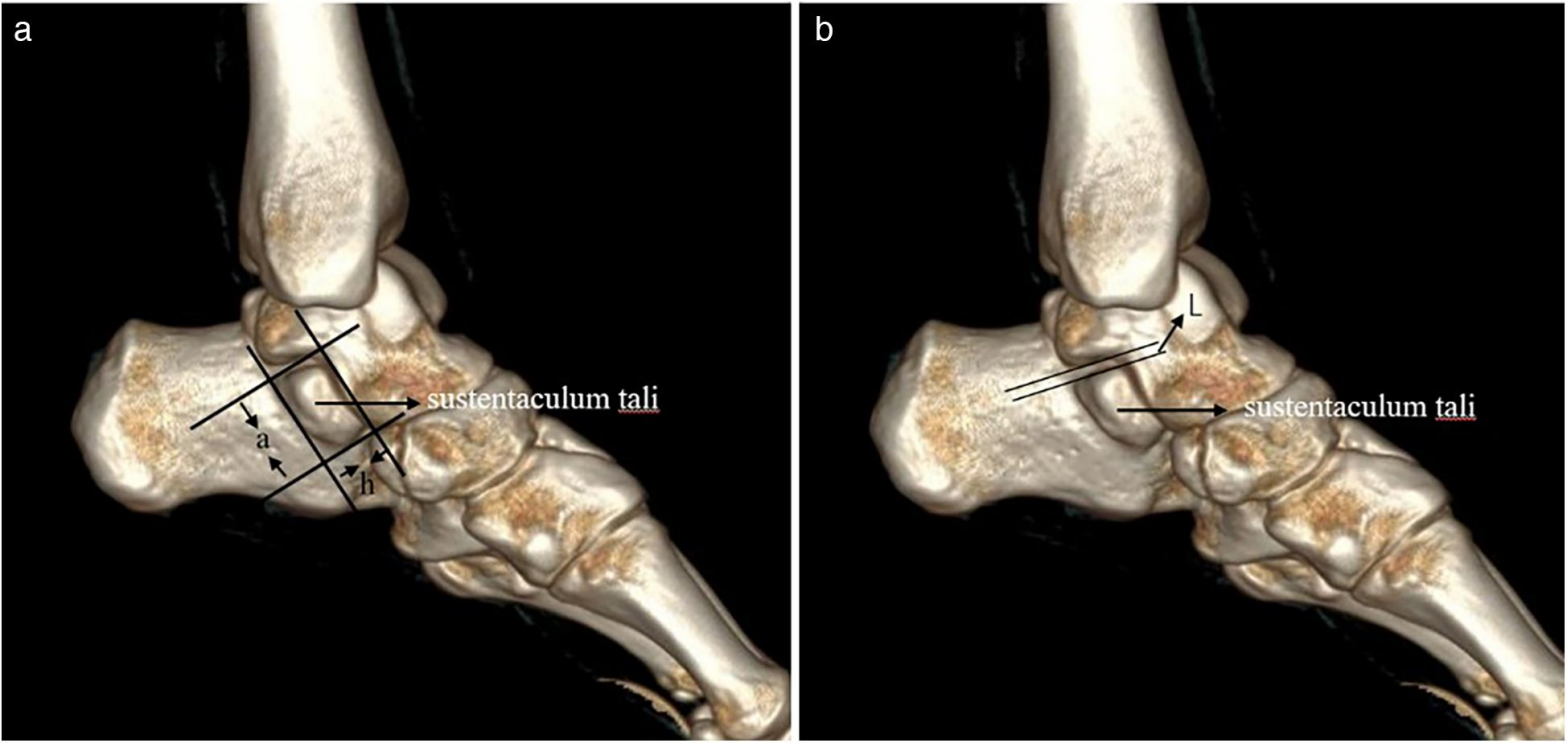
The length (a) and height (h), as well as the distance L between the posterior edge of the sustentaculum tali and the lower edge of the talus. **(a)** On the sagittal plane, the length (a) and height (h) of the loaded talus process were measured along the direction of the loaded talus bone block. **(b)** On the sagittal plane, the distance between the projection line of the posterior edge of the carrying talus and the projection line of the lower edge of the talus was measured, which was L.

**Figure 3:**
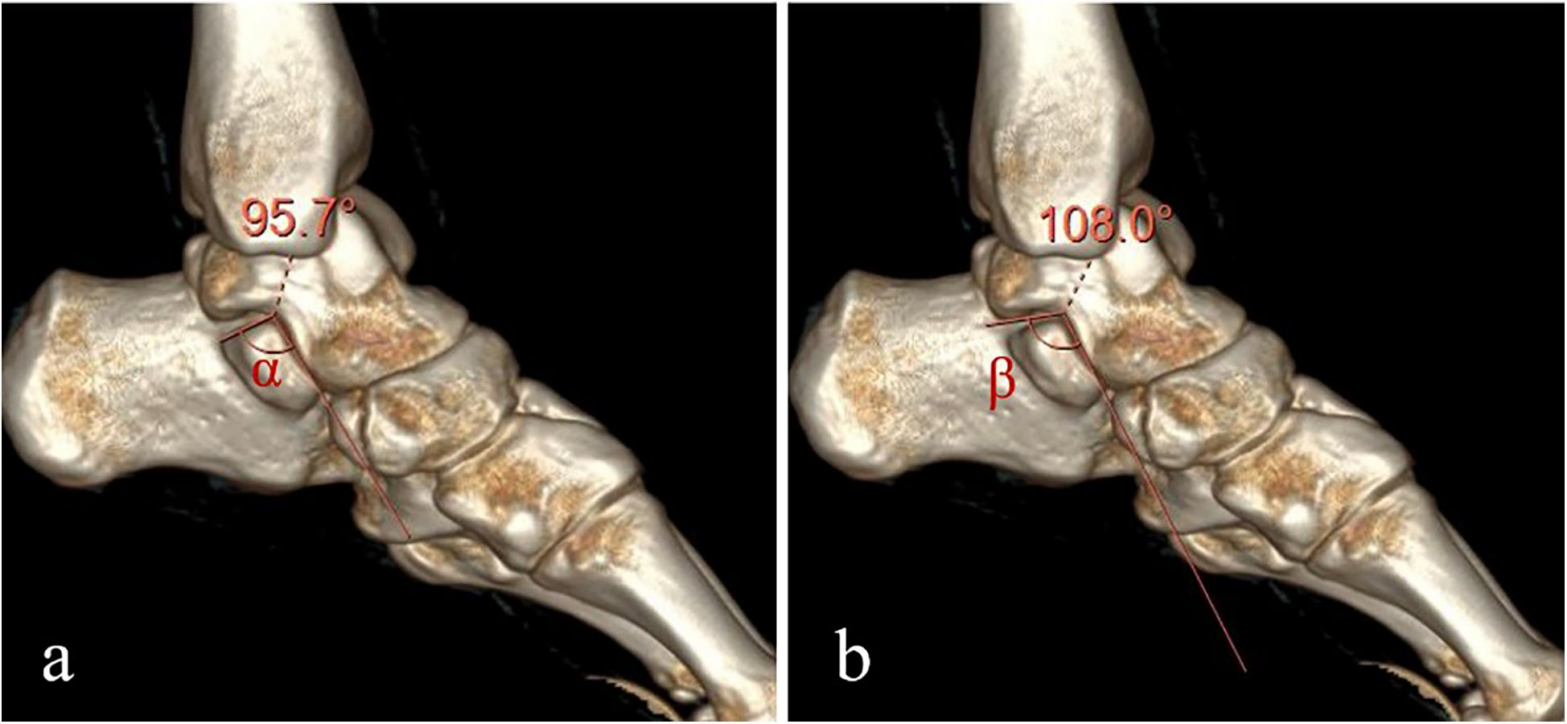
The angle α above the posterior margin of the sustentaculum tali and the angle β below the lower margin of the talus. **(a)** On the sagittal plane, the Angle between the two projection lines at the posterior edge of the sustentaculum talus, namely the Angle α above the posterior margin of the sustentaculum talus, was measured. **(b)** On the sagittal plane, the Angle between the two projection lines at the lower edge of the talus was measured, which was the Angle β below the lower margin of the talus.

**Figure 4:**
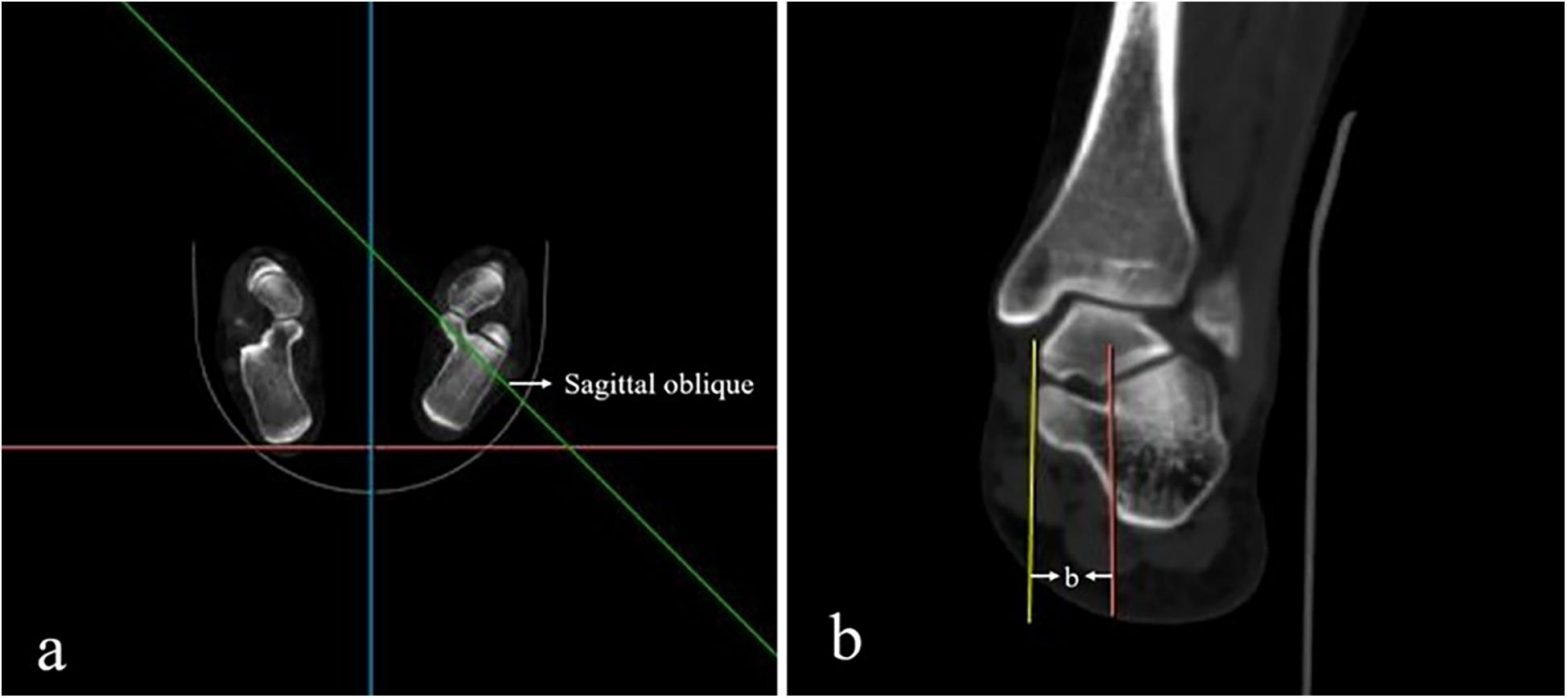
The width of the sustentaculum tali (b). **(a)** On the horizontal plane, the oblique sagittal projection line through the sustentaculum talus was found, and the multiplanar reconstruction was performed. **(b)** On the oblique sagittal plane of the multiplanar reconstruction, the distance between the projection lines on both sides of the sustentaculum talus was measured, which was the width of the sustentaculum talus (b).

**Figure 5:**
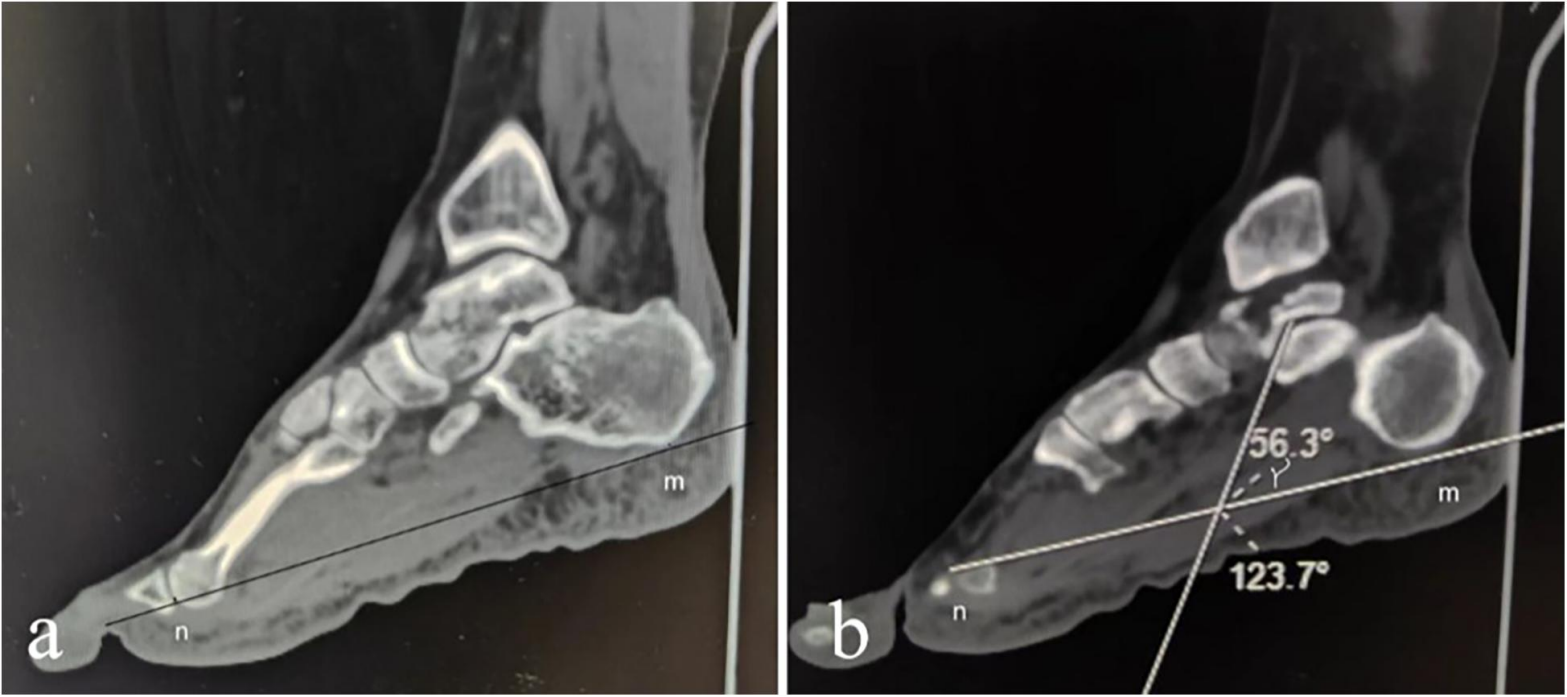
The inclination angle γ. **(a)** On the sagittal plane of multi-plane reconstruction, locate the base of the first metatarsal point m and the base of the calcaneus point n, and mark these points at various levels. Connect the mn markers to establish the projection axis of the plantar surface on the sagittal plane. **(b)** Determine the projection line of the anterior and inferior articular surface on the sagittal plane of the trochanter and calculate the angle between this projection line and the plantar axis as the inclination angle γ.

**Figure 6:**
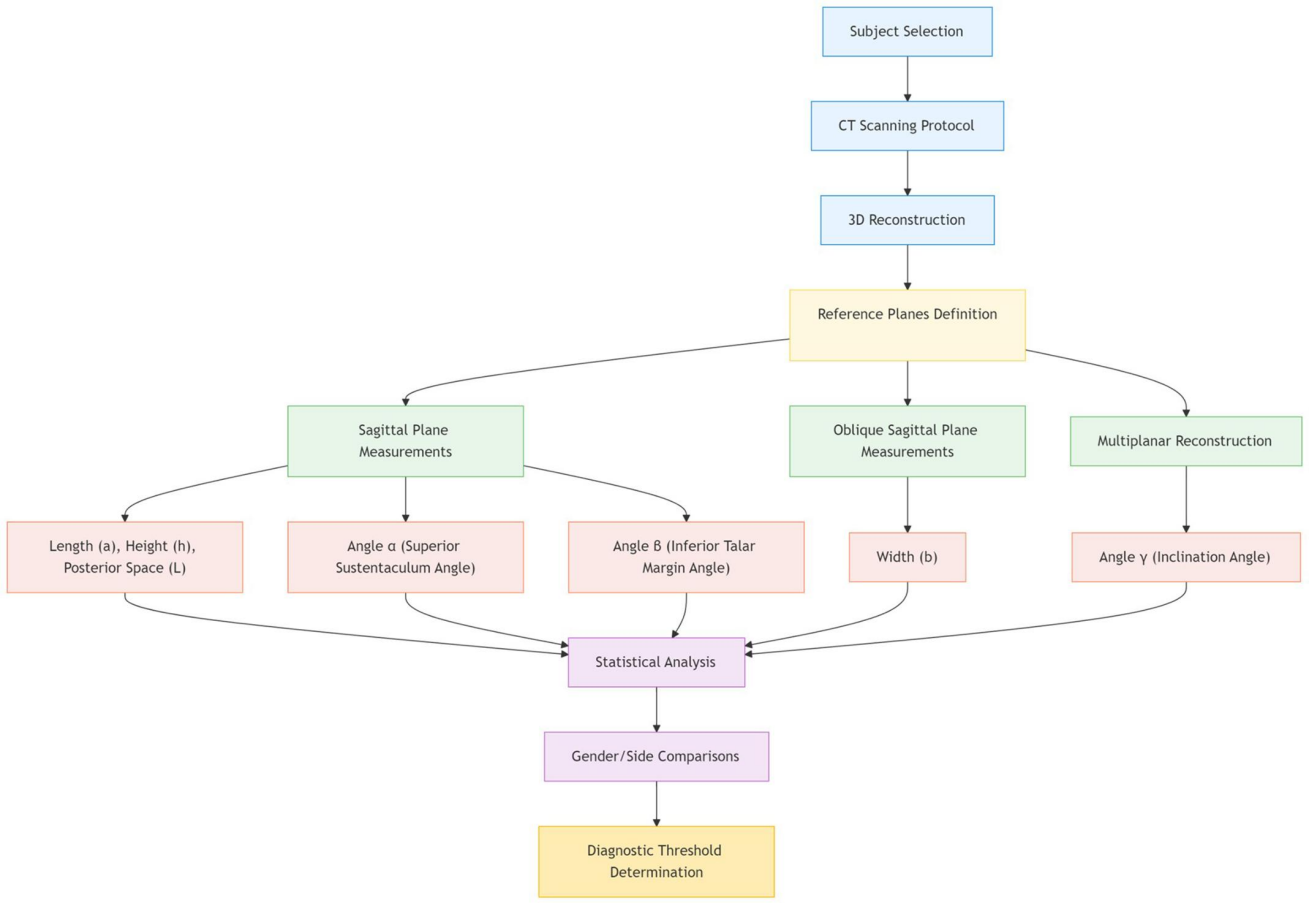
Flowchart of 3D CT-based anatomical measurement protocol for sustentaculum tali morphometry.

The morphometric parameters of the sustentaculum tali, encompassing its length (a) and height (h), in addition to the distance L from the posterior margin of the sustentaculum tali to the inferior border of the talus, were delineated and measured within the sagittal plane of the three-dimensional reconstruction imagery (Figure 2). Furthermore, the angles *α* and *β* were quantified within this plane, with angle *α* being measured above the posterior margin of the sustentaculum tali, and angle *β* below the inferior margin of the talus (Figure 3). Moreover, a refined measurement of the width of the sustentaculum tali (b) was achieved through multiplanar reconstruction employing an oblique sagittal plane orientation (Figure 4). In the multiplanar reconstruction of the sagittal plane, the m point, located at the base of the first metatarsal phalanx, and the *n* point, situated at the base of the calcaneus, were identified respectively. Subsequently, points at varying levels were demarcated, and the mn marker points were connected to establish the projection axis of the plantar surface within the sagittal plane. The projection line of the inferior articular surface of the Sustentaculum tali was then determined in the anterior sagittal plane. The angle formed by the intersection of this projection line with the plantar axis represents the angle of inclination, denoted as *γ* (Figure 5). The specific process is shown in the figure below (Figure 6).

### Statistical analysis

IBM SPSS27.0 statistical software was used, measurement data were normally distributed by Kolmogorov–Smirnov test, and measurement data with normal distribution and homogeneity of variance were expressed as $\bar{x}\pm \mathrm{SD}$. Comparisons between males and females, as well as between the left and right feet, were conducted using independent samples *t*-tests. A two-sided significance level of *α* = 0.05 was employed, with *P* < 0.05 deemed statistically significant.

Reliability was assessed using intraclass correlation coefficients (ICCs) to quantify relative reliability, encompassing both intra-observer and inter-observer agreement. Intra-observer ICCs were calculated based on repeated measurements of the same sample (*n* = 30) by a single observer at intervals exceeding two weeks, using an absolute agreement definition and a two-way random-effects model. Inter-observer ICCs were calculated based on measurements of identical samples by two independent observers, also using absolute agreement and a two-way random-effects model. Generally, ICC is considered good for 0.75–0.90 and excellent for 0.91–1.00 (15).

## Results

### Anatomical data

Morphometric analysis revealed the following anatomical parameters of the sustentaculum tali: length 36.93 ± 4.30 mm, width 14.65 ± 1.96 mm, and height 17.91 ± 2.34 mm. Angular measurements demonstrated *α* = 107.74 ± 9.59°, *β* = 115.34 ± 9.41°, and *γ* = 52.67 ± 5.98°, with posterior talar space distance L measuring 4.83 ± 0.56 mm. Gender-based comparisons showed statistically significant differences in three-dimensional sustentaculum tali morphology (length/width/height) and posterior space L (all *P* < 0.001), with female measurements being consistently smaller than males (Table 1). No significant inter-gender differences were observed in angular parameters (α/β/γ, *P* > 0.05, Table 1). Laterality analysis revealed no statistically meaningful variations between left and right feet across all measured parameters (*P* > 0.05, Table 2).

**Table 1:** Comparison of anatomical data of talar joint between male and female (mm, $\bar{x}\pm \mathrm{sd}{/}^{\circ }$, $\bar{x}\pm \mathrm{sd}$).

Gender	Foot count	Long	Wide	High	L	α	β	Γ
Male	47	38.66 ± 4.01	15.69 ± 1.39	19.25 ± 2.05	5.25 ± 0.42	106.60 ± 9.52	114.61 ± 8.36	51.48 ± 5.52
Female	57	34.96 ± 3.81	13.80 ± 1.96	16.80 ± 1.98	4.49 ± 0.41	108.68 ± 9.62	115.94 ± 10.23	53.64 ± 6.23
*t*		4.814	5.567	6.184	9.409	1.099	0.719	1.85
*P*		<0.01	<0.01	<0.01	<0.01	0.274	0.474	0.067

**Table 2:** Comparison of anatomical data of different feet (mm, $\bar{x}\pm \mathrm{SD}{/}^{\circ }$, $\bar{x}\pm \mathrm{SD}$).

Foot	Foot count	Long	Wide	High	L	α	β	Γ
Right	53	36.78 ± 4.62	14.49 ± 1.86	18.19 ± 2.30	4.86 ± 0.62	108.27 ± 10.10	114.97 ± 9.49	53.69 ± 4.87
Left	51	36.47 ± 3.97	14.81 ± 2.06	17.61 ± 2.44	4.81 ± 0.49	107.20 ± 9.09	115.72 ± 9.41	51.61 ± 6.85
*t*	–	0.371	−0.840	1.267	0.461	0.567	−0.406	1.789
*P*		0.711	0.403	0.208	0.646	0.572	0.686	0.077

### Measurement reliability

To ensure generality and accuracy, we calculated intra- and inter-observer intraclass correlation coefficients (ICCs), excluding outliers and missing values from the analysis. All parameter groups demonstrated excellent reliability, with ICCs exceeding 0.750 (Table 3), indicating strong agreement both within and between observers.

**Table 3:** Reliability analysis of sustentaculum tali parameters (*n* = 30).

Parameter	Inter-observer ICC (95% CI)	Intra-observer ICC (95% CI)	Reliability level
Length (a)	0.879 (0.761–0.940)	0.825 (0.628–0.918)	Good
Width (b)	0.900 (0.801–0.951)	0.935 (0.869–0.969)	Excellent
Height (h)	0.827 (0.666–0.914)	0.902 (0.805–0.952)	Good/Excellent
Posterior talar gap (L)	0.881 (0.765–0.942)	0.882 (0.766–0.942)	Good
Angle (α)	0.778 (0.557–0.892)	0.860 (0.729–0.930)	Good
Angle (β)	0.836 (0.644–0.923)	0.921 (0.825–0.963)	Good/Excellent
Angle (γ)	0.923 (0.846–0.963)	0.914 (0.825–0.958)	Excellent

## Discussion

### Relevance of the anatomy and biomechanics of the talar joint and sustentaculum tali in clinical practice

There exist a multitude of complex methodologies for delineating and classifying the calcaneus. Guang-rong Yu et al. (7) categorized the calcaneus into five parts: the anterior part, the body part, the colliculus part, the sustentaculum part, and the tubercle part, providing a more detailed description of its anatomical location. The anterior aspect of the calcaneus demonstrates a relatively consistent anatomical morphology, wherein the lateral wall is approximately oriented along the sagittal plane. The superior region of the calcaneocuboid joint is composed of a rugged corticated bone that slopes from the medial superior to the lateral inferior direction. The posterior upper section of its medial surface extends seamlessly into the anterior aspect of the sustentaculum tali while forming a joint with cuboid bone at its front end. The sustentaculum tali constitutes the medial component of the calcaneus, projecting above its anterior portion and extending medially, its base runs parallel to and merges with the medial wall of the calcaneus. When viewed from the ankle's medial side, the sustentaculum tali tilts in an anteversion angle from back to front. Its sole articular surface is a mid-talar articular surface that supports the talus and forms a mid-talar joint with it; a firm joint capsule attach around this area connecting the talus and calcaneus through ligaments. The medial interosseous ligament and medial talocalcaneal ligament exhibit relative toughness whereas the lateral talocalcaneal ligament is comparatively weaker; during traumatic events, interosseous ligament and medial talocalcaneal ligament are seldom damaged while the lateral one easily tears—an important factor contributing to the stability of superior-medial fracture fragment housing Sustentaculum tali during calcaneus fractures.

The increasing prevalence of high-energy skeletal trauma, compounded by the heightened complexity of these injuries and the intricate anatomy of the calcaneocuboid joint, has necessitated that orthopedic surgeons prioritize anatomical reduction and stable fixation as the quintessential goals in the surgical treatment of calcaneal fractures. In the course of surgical intervention, to guarantee robust fixation of the posterior calcaneal articular surface, it is essential to delineate a stable support point within the medial and lateral cortices of the calcaneus. The intact and relatively nondisplaced Sustentaculum tali within the medial aspect serves as an optimal anatomical landmark for this objective. Consequently, the stable anatomical structure of the Sustentaculum tali is a pivotal foundation for the efficacy of treatment in calcaneal fractures. The lateral approach is the primary surgical method for calcaneal fractures (16). However, due to the location of the sustentaculum tali on the medial side of the calcaneus, it cannot be directly observed from the lateral incision. Additionally, numerous blood vessels and nerves are running through this area, further complicating the surgical procedure. Precision in the implantation of screws into the sustentaculum tali is essential for mitigating complications including marginal incisional skin infection and necrosis, tarsal sinusitis, subtalar arthrofaciitis, and complete failure of bony synthesis (17, 18). The accurate placement of screws within the trochlear process is pivotal in the effective management of calcaneal fractures (19, 20). Therefore, comprehending the diverse anatomical morphotypes and biomechanical properties of the sustentaculum tali is crucial for determining the appropriate screw placement during surgical intervention for calcaneal fractures, and it furnishes a solid theoretical foundation for their therapeutic management.

It is noteworthy that the length, width, and height of the sustentaculum tali were found to be significantly shorter in females compared to males (*P* < 0.01, Table 1), indicating gender-specific clinical criteria for the anatomical location of the sustentaculum tali. However, other anatomical data did not show statistically significant differences between genders (*P* > 0.05, Table 1). Additionally, there were no statistically significant differences in anatomical data between different feet (*P* > 0.05, Table 2), suggesting that fracture reduction treatment plans can be based on the healthy side of the patient's foot. Furthermore, refining anatomical data related to the sustentaculum tali is beneficial for determining screw thickness and length, insertion angle, and enhancing screw insertion guide accuracy (21).

### Common clinical lesions of the sustentaculum tali

Isolated sustentaculum tali fractures are rare in calcaneal fractures, and there is limited literature on sustentaculum tali fractures. These fractures typically occur following a fall from height, with the foot in varus plantar flexion experiencing longitudinal shear force or pressure. Due to the strong attachment of the deltoid ligament around the sustentaculum tali fracture, displacement is uncommon after fracture, presenting only as swelling and pain under the medial malleolus. Diagnosis can be challenging with x-ray examination and may require three-dimensional reconstruction of CT scans, especially for simple non-displaced sustentaculum tali fractures. Previous beliefs about stability due to ligament maintenance have been challenged by studies showing a high incidence of calcaneal fracture combined with sustentaculum tali fracture, dislocation, or subluxation (12–14). Clinical studies have also indicated that a significant proportion of sustentaculum fracture fragments exhibit an Angle >10° and displacement >3 mm, with some involving the middle articular surface (12, 22).

By quantifying the width of the sustentaculum tali and analyzing the pressure exerted on the talus, several studies (23, 24) have indicated that the sustentaculum tali plays a crucial role in the subtalar joint as well as serving as a significant longitudinal weight-bearing structure. Most of the longitudinal pressure on the talus is transmitted to the sustentaculum tali. Consequently, when subjected to longitudinal shear force or pressure, fractures involving the sustentaculum tali often also involve fractures of the talus (25).

### The clinical significance of the posterior talar gap L of the sustentaculum tali

The dislocation of the sustentaculum tali in clinical practice has been underreported. Gitajn et al. (22) conducted a comprehensive analysis of 212 patients with calcaneal fractures and found that 44.3% had sustentaculum tali fractures, 20.3% had sustentaculum tali-talus subluxation, and 0.9% had complete dislocation. Diagnosis of sustentaculum talar dislocation or subluxation is primarily based on quantifying the angle between the sagittal and coronal planes of the talar and calcaneal articular surfaces. An inconsistency >5° is considered a subluxation, while complete inconsistency constitutes sustentaculum talar (complete) dislocation (12). It has also been noted that there is a significant enlargement in the gap between the talus and the posterior half of the talus sustentaculum in all patients with talus-talus sustentaculum dislocation (26). However, clear diagnostic criteria for this data have not been provided in the literature. The average length of *L* (the gap between the posterior edge of the sustentaculum tali and the lower edge of the talus) was measured at 5.25 mm in males and 4.49 mm in females. Displacement was defined as the translation of the fracture fragment in any plane exceeding 2 mm, and dislocation of the sustentaculum tali was defined as a posterior space distance exceeding the normal distance of 2 mm, with dislocation being considered when the posterior space distance L exceeded 7.25 mm in males and 6.49 mm in females. Therefore, by measuring the distance L between the posterior edge of the sustentaculum tali and the lower edge of the talus in normal subjects, clinical anatomical data for diagnosing sustentaculum tali dislocation at imaging and anatomical levels can be established. It is noteworthy that there is a statistically significant difference (*P* < 0.01, Table 1) indicating that gender-specific clinical criteria should be considered for diagnosing and operating on sustentaculum tali dislocations. These gender-specific diagnostic thresholds directly inform critical surgical decisions. Preoperative planning: When posterior space distance (L) exceeds 7.25 mm (males) or 6.49 mm (females), 3D-CT confirmation of sustentaculum tali dislocation mandates open reduction. Crew selection: The normative width (14.65 ± 1.96 mm) indicates optimal screw diameters of 3.5–4.0 mm to avoid cortical breach, while length measurements (36.93 ± 4.30 mm) guide screw sizing (typically 30–40 mm). Intraoperative guidance: Angular parameters (α/β/γ) assist in trajectory planning for sustentaculum screw placement via lateral approaches, reducing neurovascular injury risk. Collectively, these data enable surgeons to objectively determine reduction urgency, implant specifications, and approach strategies—translating anatomical insights into safer interventions.

### The clinical significance of the application of CT three-dimensional reconstruction technology

With the progression of imaging technology, CT three-dimensional reconstruction has become broadly integrated into orthopedic practice. These reconstructed 3D images offer an enhanced depth perception, enabling a vivid and detailed depiction of joint-involved fractures as well as intricate fracture patterns (27). The workstation software facilitates the selection of the bone window, effectively eliminating soft tissue shadows, allowing for the observation of any specified part and plane. It enables precise localization and measurement of any line segment and angle with a high degree of accuracy, to within 0.1 mm and 0.1°, respectively. Establishing a three-dimensional model using CT images facilitates more convenient and accurate measurement of anatomical data for the carrying talar bone block from the spatial three-dimensional position and intrinsic bone characteristics. Magnan et al. (28) contend that spiral CT surpasses conventional x-ray imaging in the context of calcaneal fractures. They argue that traditional x-ray examinations are limited to providing only two planes of imaging—axial and lateral—whereas the calcaneus exhibits a complex, irregular morphology and forms articular surfaces with adjacent bone fragments. This highlights the limitations of traditional x-ray techniques and the challenge of acquiring a comprehensive assessment of the fracture. Fa-jiao Xiao et al. (29) introduced the concept of the talar bone block via CT-generated three-dimensional imaging, thereby extending the zone of reliable anchorage from the medial calcaneus to the talar bone block. They delineated the projectional boundaries of the talar bone block on the lateral aspect of the calcaneus and quantified the dimensions of the screw insertion trajectory, thereby offering valuable referential data for the surgical management of calcaneal fractures.

### Validation of measurement reliability

Although manual measurements on 3D reconstructions introduce inherent subjectivity, our rigorous reliability analysis demonstrates consistently high reproducibility. All ICC values exceeded 0.75 (Table 3), satisfying the >0.75 threshold for clinical applications (15). Particularly noteworthy is the good inter and intra-observer agreement for posterior talar gap L (ICC = 0.881/0.882), which directly supports its use as a diagnostic criterion for dislocation.

### Limitations of this study

1. This study was limited to an analysis of 104 normal calcaneal CT scans, which may not comprehensively encompass the full spectrum of anatomical variations within the calcaneus.
2. The inherent challenge of subjective bias associated with manual landmarking during the measurement of three-dimensional images is an issue that necessitates attention in subsequent research endeavors.
3. The participant cohort was recruited exclusively from a single Chinese area. While this provides foundational data for the studied population, future multi-ethnic studies are warranted to evaluate potential anatomical variations across diverse geographic and ethnic groups.

## Conclusion

This study establishes comprehensive normative anatomical parameters of the sustentaculum tali and its spatial relationship with the talus using three-dimensional CT in a healthy cohort. Key findings include: (1) Gender-specific diagnostic thresholds for sustentaculum tali dislocation (posterior space distance *L*>7.25 mm in males; >6.49 mm in females), enabling urgent surgical intervention when exceeded; (2) Dimension-based screw selection guidance, where normative width (14.65 ± 1.96 mm) supports 3.5–4.0 mm screw diameters to prevent cortical breach, and length (36.93 ± 4.30 mm) informs 30–40 mm screw sizing; (3) Angular parameters (α/β/γ) for trajectory planning during lateral-approach screw placement, reducing neurovascular risks; (4) Absence of laterality differences, permitting contralateral-foot templating for unilateral fractures. Supported by excellent measurement reliability (ICCs >0.75), this anatomical framework transitions calcaneal fracture management from empirical to evidence-based precision. Future validation in multi-ethnic clinical cohorts will be essential to assess anatomical variability across populations and establish universally applicable protocols.

## Data Availability

The raw data supporting the conclusions of this article will be made available by the authors, without undue reservation.
